# Are Patients With *Schizophrenia Spectrum Disorders* More Prone to Manifest Nocebo-Like-Effects? A Meta-Analysis of Adverse Events in Placebo Groups of Double-Blind Antipsychotic Trials

**DOI:** 10.3389/fphar.2019.00502

**Published:** 2019-05-17

**Authors:** Sara Palermo, Fabio Giovannelli, Massimo Bartoli, Martina Amanzio

**Affiliations:** ^1^Department of Psychology, University of Turin, Turin, Italy; ^2^European Innovation Partnership on Active and Healthy Ageing, Brussels, Belgium; ^3^Section of Psychology, Department of Neuroscience, Psychology, Drug Research, Child Health, University of Florence, Florence, Italy

**Keywords:** schizophrenia, randomized clinical trials, placebo, nocebo effects, adverse events, atypical antipsychotic drugs, expectation theory

## Abstract

**Background:** Antipsychotic clinical trials use to present adverse events (AEs) for the drug under evaluation to treat schizophrenia. Interestingly, patients who receive the placebo during antipsychotic trials often report several AEs, but little is known about the essence of these negative effects in patients with schizophrenia spectrum disorders (SCD). In the present meta-analysis, we evaluated the relationship between the level of psychiatric symptomatology expressed as Positive and Negative Syndrome Scale (PANSS) scores and the rates of AEs reported in the placebo arms of double-blind clinical trials, for commonly prescribed atypical antipsychotic medications.

**Methods:** We selected 58 clinical trials describing AEs in SCD placebo groups, which compared atypical antipsychotic medications with placebo. A total of 6,301 placebo-treated patients were considered. AE profiles of the class were clusterized using MedDRA classification and analysed using a meta-regression approach.

**Results:** In the placebo arms the proportions of patients with any AE was 66.3% (95% CI: 62.7–69.8%). The proportion of withdrawal of patients treated with placebo because of AEs was 7.2% (95% CI: 5.9–8.4%). Interestingly, the AEs in the placebo arms corresponded to those of the antipsychotic-atypical-medication-class against which the placebo was compared. Namely, using meta-regression analysis we found an association between the level of psychiatric symptomatology measured with PANSS scores and higher AEs reported as nervous system (*p* = 0.020) and gastrintestinal disorders (*p* = 0.004). Moreover, the level of a higher psychiatric symptomatology expressed with PANSS scores was also related with higher AEs associated with psychiatric symptoms (*p* = 0.017).

**Conclusion:** These findings emphasise that the AEs in placebo arms of clinical trials of antipsychotic medications were substantial. Importantly, a higher level of psychiatric symptomatology makes SCD patients more prone to express AEs, thus contributing to possible drop-outs and to a lower adherence to treatments. These results are consistent with the expectation theory of placebo and nocebo effects.

## Introduction

Adverse events (AEs) in pharmacological treatments of psychiatric disorders are something that significantly affect the adherence and the drop-outs of patients (Wahlbeck et al., 2001). Many AEs are directly derived from the specific pharmacological actions of an antipsychotic drug. It is well-known that different peculiar AEs are caused by different specific antipsychotics (Stroup and Gray, 2018). For example, in the treatment of schizophrenia spectrum disorders (SCD), different pharmacological agents can be used. In recent years clinicians have progressively treated schizophrenia using atypical (or second-generation) antipsychotics in preference to “conventional” typical (first-generation) drugs (Crossley et al., 2010). These two different class of drugs induce different rates of AEs. Typical antipsychotic drugs are associated with higher risk for extrapyramidal symptoms (EPS) and cognitive impairment. In contrast, atypical forms show better adverse event profiles and have a lower incidence of drug-induced EPS, compared with the other class (Lehman et al., 2004). However, patients treated with atypical drugs frequently report a number of AEs such as: seizures, sedation, orthostatic hypotension, anticholinergic effects, weight gain, prolactin increases, hepatic changes, and agranulocytosis (Meltzer, 1998).

Hwang et al. (2010) suggested that unfavorable treatment related expectation effects ≪might result in certain psychological and somatic symptoms (Barsky et al., 2002; Benedetti et al., 2006; Reeves et al., 2007), a phenomenon largely studied as nocebo effect…If the nocebo concept for the understanding of the interaction between psychopathology and subjective side effects is adopted, it could be hypothesized that certain psychopathological conditions might make patients more prone to a negative treatment expectation, which may in turn adversely affect their subjective experience and evaluation of side effects≫ (pp. 83–84). It has been seen that depression, anxiety, and a tendency to somatize (Barsky et al., 2002), seem to be linked with the nocebo phenomenon observed in placebo groups of RCTs and/or through the reports of non-specific side effects with active medications (Hwang et al., 2010). It is plausible that the level of psychopathology in patients with psychiatric disorders widely affected their perceptions and attribution of the bodily sensations toward medications (Hwang et al., 2010); however, to our knowledge, no studies have investigated how that turns into the subjective report of AEs in patients with SCD, using a meta-analysis approach. Interestingly, only one study investigated the relationship between psychopathology and subjective AEs showing that self-reported side effects in patients with schizophrenia may be influenced by the severity of positive symptoms, and signs of anxiety and depression. Essentially, the results of the study indicated that positive symptoms not only directly affect subjectively reported AEs, but also affect them indirectly by influencing anxiety symptoms (Hwang et al., 2010). The authors concluded that schizophrenic patients, with high levels of positive and anxiety/depressive symptoms, may be more susceptible to nocebo-like-effects of antipsychotics.

In a classic clinical trial, participants know they can receive either the active medication or the placebo and, accordingly, they are informed about the possible AEs they may experience during the trial (Amanzio et al., 2016). Different studies on both placebo and nocebo effects have shown that expectations about the therapeutic outcome play a critical role in the response to treatment (Kirsch, 1985; Amanzio et al., 2001; Benedetti, 2008; Enck et al., 2008; Price et al., 2008). This applies to both positive suggestions leading to positive outcomes and negative suggestions bringing to a worsening of symptoms, as in the case of nocebo-like-effects. Therefore, informing subjects about the possible AEs they may experience, may have a significant impact on their expectations of negative effects (Amanzio et al., 2016). In line with these considerations, we conducted the first study that analysed randomized clinical trials of three different classes of anti-migraine medications (non-steroid anti-inflammatory drugs, triptans and anticonvulsants) that reported rates of AEs in placebo groups (Amanzio et al., 2009). We demonstrated that the AEs in the placebo groups corresponded to those of the anti-migraine medication against which the placebo was compared. Moreover, the number of AEs in the placebo arms of trials with anti-migraine drugs was high. Similar results were obtained in a systematic review that considered nocebo effects for antidepressant placebos comparable to the AE-profiles of different classes of tricyclic antidepressants (Rief et al., 2009). These key results are not consistent with the belief that nocebo effects were simply non-specific.

The present study represents the first meta-analysis of nocebo-like-effects in SCD patients. We evaluated AEs in placebo treated patients in RCTs of atypical antipsychotic drugs. Our hypothesis was that the placebo-related-AEs may corresponded to those associated at the atypical antipsychotic class. Moreover, we considered the role of the level of psychiatric symptomatology in experiencing AEs. Namely, we expected increased rates of AEs associated to higher scores of the Positive and Negative Syndrome Scale (PANSS: Kay et al., 1987). Indeed, as an objective measurement of psychopathology, PANSS offers an assessment of a comprehensive range of symptom domains in SCD through positive, negative and general symptoms subscales. We hypothesized that the severity of illness may make patients more prone to developing distressing symptoms related with treatments.

## Materials and Methods

### Study Selection

**Figure 1:**
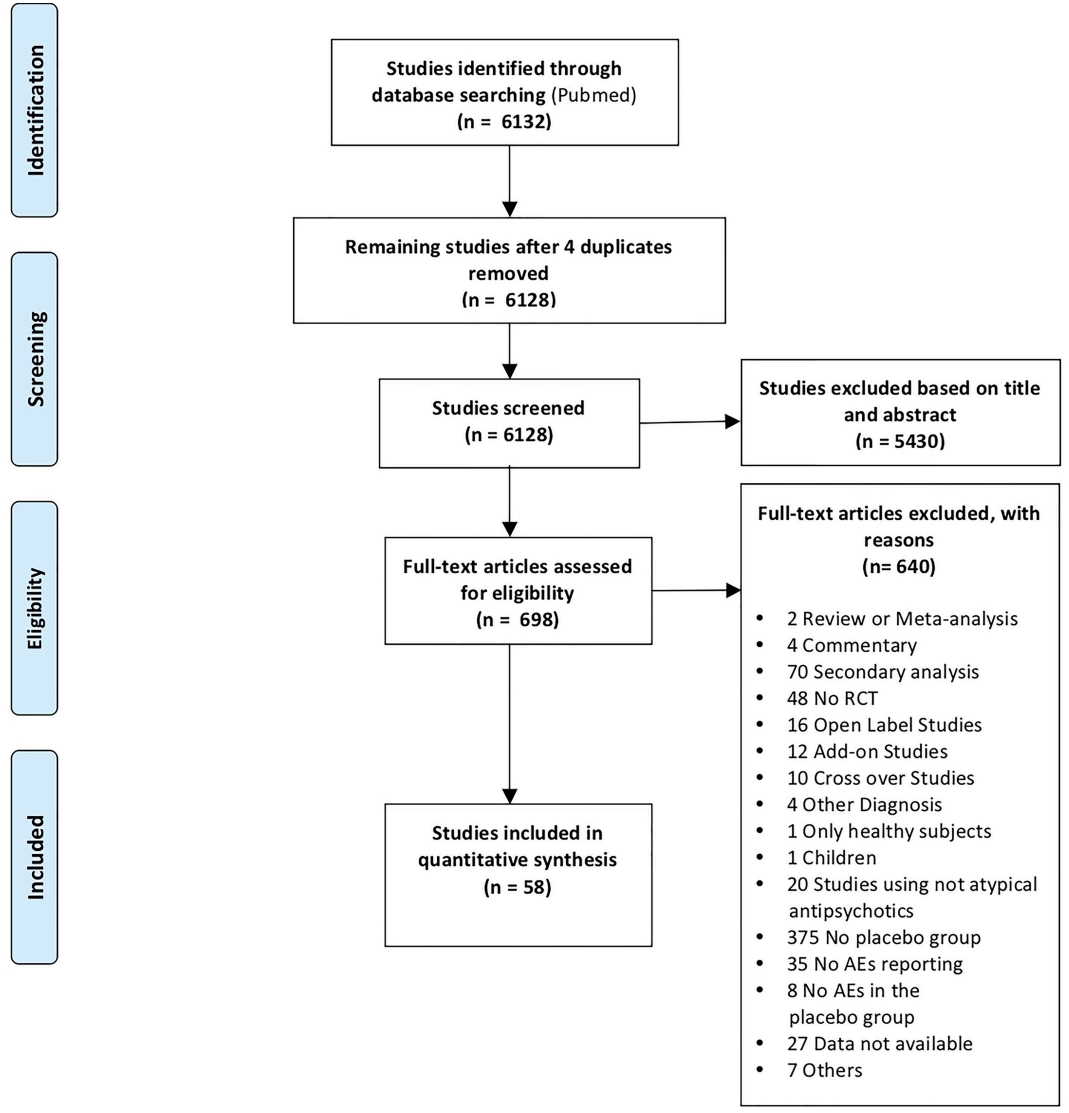
PRISMA flow chart.

We embraced the definition of meta-analysis accepted by the Cochrane Collaboration (Green et al., 2008) and the “PRISMA Statement” international guidelines in order to guarantee a see-through report of our data selection procedures (Liberati et al., 2009; Moher et al., 2009). A systematic search strategy was implemented to identify relevant SCD studies, published until 1 March 2018, across the online database most frequently used in the international literature (Medline database with PubMed literature search: http://www.ncbi.nlm.nih.gov/pubmed). All the articles that did not meet the inclusion criteria were excluded. With this aim, we reviewed the relevant literature in order to ensure: (1) the inclusion of either the healthy control group and the pathological sample (patients with SCD); (2) randomized, double-blind, placebo-controlled and parallel design; (3) administration of commonly prescribed atypical antipsychotic medications or experimental drugs with an atypical antipsychotic profile for SCD; (5) report of placebo related AEs; (6) the original diagnosis made on the basis of DSM criteria and clinical test batteries; and (7) that the studies were original works. Instances of multiple references to the same data sets across articles were identified so as to make sure that only one reference to the same data contributed to the present meta-analysis [see Table S1, Appendix 1 in the Supporting Information and Prisma Flow Chart in the main text (Figure 1)].

Specifically, three of the authors screened titles/abstracts of all identified articles. Subsequently, two authors authors examined the full texts independently (double-blind) and according to the pre-defined eligibility criteria. The full text that have not been found were requested by mail. The use of the same investigators to accomplish selection ensured both consistency between the selected studies and the only inclusion of RCTs that optimally defined the target population of patients and the placebo arm. Significantly, investigators who carried out this research stage have reached substantial agreement (% of agreement 96.50655; Cohen's K 0.50192).

While we were in the selection phase, we found few studies that analysed placebo treated patients in RCTs of typical antipsychotic drugs, this unfortunately made it impossible to compare the two pharmacological classes (typical and atypical) considering the respective placebo groups.

Two authors independently conducted data-extraction and performed the assessment of methodological quality. Disagreements that occurred in these phase were solved through discussion between all authors. The Cochrane Collaboration's tool for assessing risk of bias (Higgins and Altman, 2008; Higgins et al., 2011) was used to ascertain the validity of eligible studies. Six domains were assessed for each study: (1) sequence generation (selection bias); (2) allocation concealment (selection bias); (3) blinding of personnel and outcome assessors (performance bias); (4) blinding of outcome assessment (detection bias); (5) incomplete outcome data (attrition bias); and (6) selective outcome reporting (reporting bias). The domains “incomplete outcome data” and “selective outcome reporting” were separately assessed for patients withdrawing because of AEs and occurrence of AEs (including serious AEs).

Considering the pharmacological treatment, drugs considered eligible to enter in our database were: Amisulpride, Aripiprazole, Asenapine, Bifeprunox, Brexpiprazole, Cariprazine, Fananserin, Iloperidone, Lumateperone (ITI-007), Lurasidone, Olanzapine, Paliperidone, Quetiapine, Risperidone, Sertindole, Vabicaserin, Ziprasidone, and Zotepine. Studies comparing two different classes of anti-psychotic drugs (i.e., typical and atypical) in the same trial were excluded.

### Outcomes Measures Search

Outcome measures were retrieved from tables reporting AEs or from the main text of each selected original article. Data were extracted in the same way as it has been done in previous meta-analyses by our group (Amanzio et al., 2009; Giovannelli et al., 2015; Zaccara et al., 2015). Briefly, for each study we extracted: (a) the number of randomized patients (intent-to-treat population, ITT); (b) the total number of AEs reported in placebo treated patients (c) the number of patients withdrawing because of AEs and PANSS score. AEs were categorized into three groups based on MedDRA classification (Brown et al., 1999) codified as: (1) nervous system disorders; (2) psychiatric disorders and (3) gastrointestinal disorders. We considered few terms as synonymous of the considered AEs. For details on synonyms, see Table S2.

### Data Analysis

Statistical analyses were performed using SPSS version 25.0 (IBM Corp, 2017) for Windows. Data for the principal demographic and clinical characteristics (PANSS) are expressed as range, percentage or the mean ± standard deviation.

Proportions of patients randomized to placebo arm with 95 % confidence intervals (CIs) have been expressed for all reported outcome measures. The meta-analysis was conducted using the software Open Meta-Analyst (Wallace et al., 2009). Heterogeneity between studies has been assessed by *I*^2^ and Cochrane Q test. A random effects model has been used due to heterogeneity among studies.

A weighted least squares meta-regression has been performed to explore the effect of multiple factors (i.e., year of publication, number of study arms, duration of study and severity of disease) on the selected outcome measures (total number of AEs, withdrawal for AEs, nervous system disorders, gastrointestinal disorders and psychiatric disorders). The mean PANSS score has been used to evaluate the relationship between disease severity and AEs. The meta-regression model has been weighted by the inverse of variance of each study. When the above-mentioned data were not found, we excluded the original research from the meta-regression analyses of that data. In the meta-regression analysis significance was set at *p* < 0.05.

## Results

### Selection Outcome

Fifty eight RCTs were included in the analysis (1 for Amisulpride, 8 for Aripiprazole, 3 for Asenapine, 1 for Bifeprunox, 3 for Brexpiprazole, 5 for Cariprazine, 1 for Fananserin, 1 for Iloperidone, 1 for Lumateperone (ITI-007), 7 for Lurasidone, 4 for Olanzapine, 12 for Paliperidone, 3 for Quetiapine, 2 for Risperidone, 1 for Sertindole, 1 for Vabicaserin, 3 for Ziprasidone, and 1 for Zotepine).

### Characteristics of Eligible Trials

The main features of the 58 RCTs selected for the statistical analyses are reported in Table 1. In the selected RCTs, 6,301 patients were treated with placebo. Sample size of patients treated with placebo varied across trials from a minimum of 22 to a maximum of 207 patients. Mean duration of all double-blind period (titration plus maintenance) (mean ± SD) was 12.78 ± 14.6 weeks (range 1–52 weeks).

**Table 1:** Characteristics of the placebo arm group (meta-sample for our meta-analysis).

	**% range**	**Mean (±SD)**	**Range**
Sample size (*n = 6301*)			22–207
Male (%)	67.6%		
Female (%)	32.4%		
Age (years)		39.78 (±5.17)	24.4–69
PANSS Total score		87.2 (±15.08)	43.1–104

*PANSS, Positive and Negative Syndrome Scale; SD, Standard Deviation*.

### Risk of Bias Validity of Eligible RCTs

**Figure 2:**
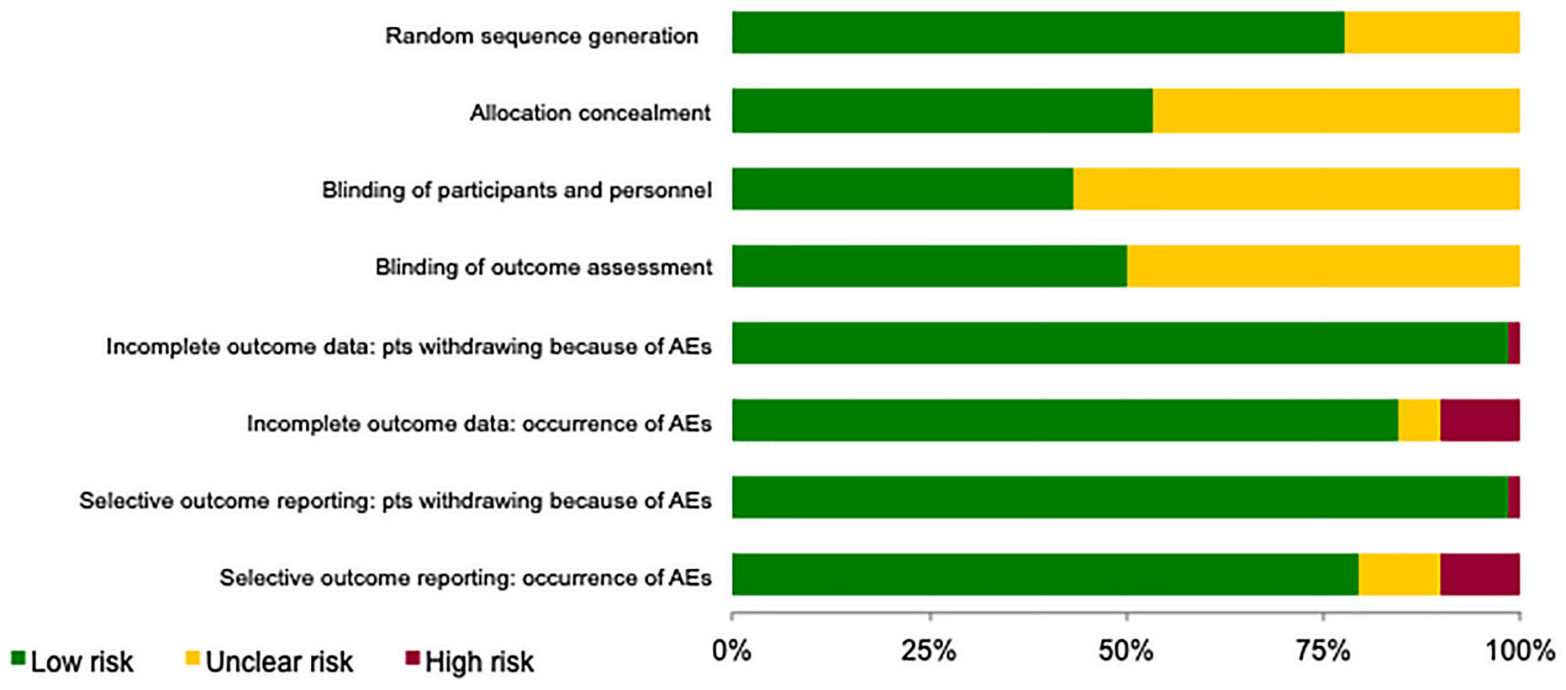
Assessment of the risk of bias in included studies. The judgement for each entry involves assessing the risk of bias as “low risk,” as “high risk,” or as “unclear risk,” with the last category indicating either lack of information or uncertainty over the potential for bias.

Risk of bias of the included studies is shown in Figure 2. For the selection bias, allocation concealment was the more poorly reported domain with 27 studies (47%) being judged to have an unclear level of risk; 13 studies (22%) were judged to have an unclear risk in the random sequence generation domain.

Regarding performance and detection biases, most information is from studies at low or unclear risk: 33 (57%) and 29 (50%) studies, respectively, were judged to have an unclear level of risk as insufficient information were provided.

For the attrition and reporting biases, separately assessed for patients withdrawing because of AEs and occurrence of AEs (including serious AEs), most information is from studies at low risk.

### Nocebo Like Effect in RCTs

Table 2 report the number of placebo-treated patients withdrawing due to AEs, the total number of patients who had experienced AEs and the number of side effects for the 3 selected classes of AEs.

**Table 2:** Proportion (95 % CI) of patients treated with placebo from 58 double-blind, placebo-controlled studies performed in patients with schizophrenia spectrum disorder for each outcome measures.

	**Number of events**	**Total number of patient in the placebo arm**	**PR (%)**	**95% CI (%)**	**Heterogeneity**	
					**I^2^**	**Cochran's Q test**
Withdrawal for AEs	518	6097	7,2	(5.9, 8.4)	84%	<0.001
All AEs	3621	5523	66,3	(62.7, 69.8)	88%	<0.001
Nervous system disorders	1596	6281	27,6	(22.9, 32.2)	97%	<0.001
Psychiatric disorders	1929	6298	30,4	(24.8, 36.0)	98%	<0.001
Gastrointestinal disorders	693	5370	12,9	(10.8, 15.0)	93%	<0.001

*AEs, Adverse Events; CI, Confidence Interval; PR, Proportion*.

Heterogeneity was generally high. In particular, the proportion of AEs reported varies between 0.02 and 17% depending on the side effect considered. Table 3 report the proportions of subjects who had experienced each of the selected AE for each of the three conditions assessed.

**Table 3:** Adverse events in the placebo arm experimental group.

	***N***
**NERVOUS SYSTEM DISORDERS**	
Akathisia	240
Ataxia	0
Attention difficulties	0
Back pain	40
Cogwheel rigidity	1
Diplopia	1
Dizziness	118
Diskynetics events	1
Dystonia	3
Epilepsy	2
Extrapyramidal disorders	91
Headache	620
Hyperkinesia/hypertonia	31
Language difficulties	0
Memory impairment	0
Myalgia	7
Pain	89
Paresthesia/tingling	18
Parkinsonism	2
Sedation/somnolence	282
Tremor	50
**PSYCHIATRIC DISORDERS**	
Abnormal thinking	295
Agitation	324
Aggressive reaction/behavior	6
Anxiety	312
Apathy	6
Depression	16
Hostility	4
Insomnia	713
Nervousness	22
Psychosis	231
**GASTROINTESTINAL DISORDERS**	
Abdominal pain	14
Diarrhea	75
Dry mouth	20
Dyspepsia	178
Nausea	182
Vomiting	178
Toothache/tooth disorders	46

*AEs, Adverse Events; N, number of patients reporting symptoms*.

In general, nervous system and psychiatric disorders AEs were significant associated with higher score on PANSS scale. Moreover, patients with a higher degree of psychiatric AE-symptoms achieved higher scores on PANSS scale (see Table 4).

**Table 4:** Results of meta-regression analysis.

	**Year of publication**		**Number of study arms**		**Duration of the study**		**PANSS Score**	
	***P***	***R***	***P***	***r***	***P***	***r***	***P***	***r***
Withdrawal for AEs	0.542	0.083	0.029	0.292	0.169	0.190	0.091	0.264
All AEs	0.308	−0.149	0.125	0.222	0.290	−0.158	0.052	0.318
Nervous system disorders	0.056	−0.255	0.499	0.091	0.521	−0.088	0.020	0.359
Psychiatric disorders	0.189	−0.177	0.005	−0.369	0.002	0.416	0.017	−0.367
Gastrointestinal disorders	0.961	−0.007	0.158	0.205	0.884	0.021	0.004	0.460

*The effect of 4 factors (year of publication; number of study arms; duration; and PANSS score) on reported outcome measures were analyzed. P-values and r coefficients are given*.

*AEs, Adverse Events; PANSS, Positive and Negative Syndrome Scale*.

**Figure 3:**
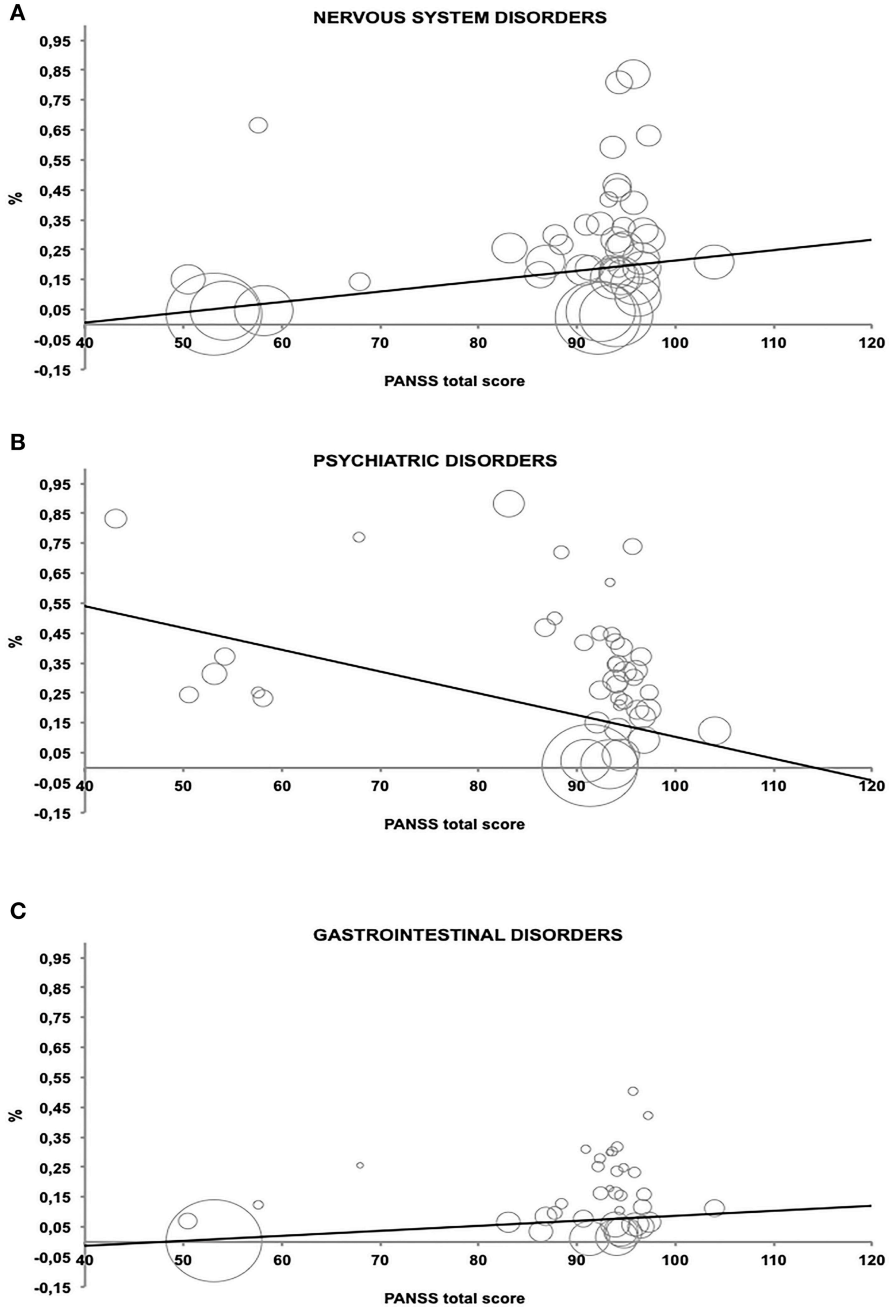
Relationship between percentage of Adverse Events and PANSS total score estimated by an inverse of variance-weighted linear meta-regression model (bubbles represent the inverse of variance). **(A–C)** which represent the AE related to the PANSS score.

The association between PANSS and the three selected AEs groups were expressed by an inverse of variance-weighted linear meta-regression model in Figure 3. Meta-regression revealed significant relationship among the symptom severity of SCD patients and the considered AEs.

## Discussion

As previously reported by Amanzio (2015), ≪in double-blind, randomized clinical trials, the AEs reported in placebo-treated groups are not associated with a pharmacological treatment, but other factors should be taken into account to explain these symptoms. This phenomenon may be conceptualized as “nocebo-like-effects” relating to negative expectations for treatment outcome, even though a role of prior learning in the form of conditioning with active treatments cannot be excluded. This approach makes it possible to observe how associating the placebo groups with a particular drug can cause specific AEs that are consistent with those observed in the active group≫ (page 159).

Before ingoing the double blind arm of a treatment trial, the examiner's instructions about the possible AEs associated with a specific antipsychotic drug may affect the outcomes (Amanzio et al., 2009). In particular, behaviors manifested by health care providers and verbal suggestions are likely to vary significantly across research contexts and may accordingly generate significant variability in nocebo-related AEs (Amanzio et al., 2016). Specifically, if the active treatment is denoted as an atypical antipsychotic, the examiner may imply that patients will experience those AEs that are in line to this class of drugs (Haddad and Sharma, 2007). For example, and considering the three drugs most represented in our meta-analysis (information obtained from *Food and Drug Administration*, FDA):

- Paliperidone: abdominal pain, back pain, blurred vision, bradykinesia, constipation, diarrhoea, dizziness, dry mouth, dystonia, fatigue, headache, hypotension, insomnia, muscular stiffness, musculoskeletal pain, nasal symptoms, nausea, parkinsonism, pharyngitis, rash, restlessness, salivation increased, irregular heartbeat, somnolence, tremor vomiting.
- Aripiprazole: constipation, diarrhoea, dizziness, headache, heartburn, increased appetite, increase salivation, pain, weight gain.
- Lurasidone: abdominal pain, anxiety, dizziness, dry mouth, extrapyramidal disorders, insomnia, muscle stiffness, nausea, rash, restlessness, salivation increased, somnolence, vomiting.

Indeed, patient's inauspicious antipsychotic-related expectations and effect might result in certain somatic and psychological symptoms. Rief et al. (2009) previously described this phenomenon through their systematic review examining placebo AEs in tricyclic antidepressant randomized clinical trials. The authors depicted nocebo effects in antidepressant placebos similar to the AE profiles of the real drugs, which they were matched with (Rief et al., 2009). Moreover, AEs may also be due to other factors such as the level of psychopathology observed in SCD patients. Interestingly, psychopathology—such as severity of depressive/anxiety or positive symptoms - adversely affected self-reports of AEs in this kind of patients (Hwang et al., 2010). Importantly, although the patients' neuropsychosocial profile is little analysed in RCTs, it is likely to be operating in nocebo effects in those patients with reduced global executive functioning (Kim et al., 2002; Hwang et al., 2009). It may be hypothesised that cognitive impairments at the level of the processes involved in the generation of precise spoken definitions, such as auditory-verbal working memory, generative ability, oral fluency and semantic retrieval (Lezak et al., 2004), may be also responsible of subjective over-generalizing discomfort. We observed these aspects in studding the placebo arms of donepezil trials (Amanzio et al., 2012). Indeed, we found Alzheimer's disease patients to be at a superior risk of developing AEs than subjects with a mild cognitive impairment.

The present study represents the first meta-analysis aiming to underline the presence of nocebo-like AEs in placebo groups matched with an antipsychotic atypical drug in the SCD treatment. We have found that 65.5% of the patients treated in placebo arms of trials described AEs and 8.5% therefore discontinued the treatment. Interestingly, our meta-regression approach demonstrated a relationship between the frequency of AEs at nervous system and gastrointestinal levels and higher PANSS score. Indeed, a higher level of psychiatric symptomatology is associated with a tendency to over-report specific AEs, thereby making patients, who suffer most from their psychiatric symptoms, more prone to developing distressing events related with treatments (Hwang et al., 2010). Therefore, patients with severe levels of depressive/ positive and anxiety symptoms may be sensible to antipsychotics nocebo-like effects, as previously shown by a study by Hwang et al. (2010). A possible explanation is that of a negative outcome risk possibly related with the presence of a chronic disease. Research works concerning the evaluation of AEs and tolerability of antipsychotics should benefit by considering the implication of these findings. Above all, it would be advisable to follow better the treatment adherence in the presence of AEs not only in the acute phase but also after symptoms stabilization.

It is worth noting that the AE profiles reported in the clinical trials presented in this meta-analysis are not influenced by possible impact factors such as the year of publication, the number of study arms and the duration of the study. Since we specifically wanted to analyse possible effects of suggestions related with research contexts, our meta-analysis did not include a trial design comparing two different classes of antipsychotic pharmacological treatment or crossover trials. Actually, the possible contribution of pharmacological conditioning in crossover RCTs could represent a confounding factor.

As previously reported by Meister et al. (2017, p. 296) some possible limitations of the meta-regression approach need to be also mentioned: (1) it may generate false-positive associations that reflect no real associations, but are caused by chance alone (Higgins and Thompson, 2004); (2) by conducting meta-regression analyses based on aggregated data of primary trials, we can only draw inferences on a study level and not on an individual level (Berlin et al., 2002; Rabinowitz et al., 2016). Meta-regression analyses on individual data may consequently result in different results; (3) it do not allow causal interpretation of the results; (4) we only conducted univariate but not multivariate meta-regression analyses due to power considerations. By fitting univariate models only, we could not simultaneously control for other modifiers; (5) the number of the included trials limit our results just like the fact that the analyzed pool of studies were heterogeneous concerning the sample clinical features such as gender and age. However, meta-regression analyses revealed hardly any association between these characteristics and the outcome rates; (6) we included only trials that investigated adults (age range 24.4–69). Certainly, our results may not be transferable to younger populations (e.g., children and adolescents). (7) Our analyses could only be dropped on the total PANSS score. This can be reductive. As previously reported by Hwang et al. (2010) some PANSS factors (delusion, hallucination, suspicion/persecution, and unusual thought content) produce misattributions of bodily sensations that directly contribute to over-generalized reporting of AEs.

An important source of bias could have emerged from the evaluation of the risk of bias (Higgins et al., 2011). Considering the purposes of our work, blinding of personnel and outcome assessors (*performance bias*) and blinding of outcome assessment (*detection bias*) are particularly important. These are also the most critical domains because in about half of the studies we have given an evaluation of “Unclear risk”. This could have led to a possible bias in the results and, consequently, in their interpretation.

We here highlight methodological shortcomings with the aim of suggesting how the detection and reporting of AEs can be improved in future RCTs. The insights from our current study should be considered when designing clinical trials to tailor individualized treatments. Our results emphasize the importance of outlining standardized detection procedures for collecting relevant data in randomized, double-blind, placebo-controlled trials of drug efficacy in patients with SCD. In particular, adequate methodology, planning and execution are critical issues in RCTs of psychiatric patients (Porter et al., 2003).

On the basis of the general nocebo effect literature of the specific disease we have analysed, it will be possible to hypothesize standardization of procedure in clinical trial designs through methodological shortcomings, which may contribute to tailoring individualized treatment: (1) appropriate assessment strategy of AEs, (2) putative patient-related psychological-psychiatric factors and baseline characteristics, and (3) behaviours and verbal suggestions manifested by medical staff and researchers. Importantly, a complex and not yet developed integrated approach to the study of these combining factors would be required to clarify the presence of psychological distress predisposing patients to report nocebo-related AEs to an even greater extent (Amanzio et al., 2012). We previously hypothesized this kind of a more accurate assessment in neurological patients with chronic disease, but these conclusions may also be applied in the psychiatric population. Specifically, as Häuser et al. stated 2012, specific strategies to reduce nocebo effects should be further developed in clinical trials and practice to minimize these symptoms (Enck et al., 2013), also considering patients with SCD. As we have previously reported (Amanzio, 2015), ≪Rief et al. (2009) rightly observed that drug trials should consider the base rates of pre-existing general complaints more rigorously in the population being studied≫ (page 160), to distinguish drug-associated AEs from the general base rates of symptoms represented by patients' characteristics in terms of significant mood changes in terms of depression and anxiety, tendency to catastrophizing, prior experiences with AEs, pre-existing symptoms and the tendency toward somatization, symptom amplification and selective attention on bodily sensations; all of which were associated with nocebo effects (Wolf and Pinsky, 1954; Andrykowski and Redd, 1987; Barsky et al., 2002; Nestoriuc et al., 2010). Moreover, neuropsychosocial factors, such as general cognitive impairment and executive dysfunction that may influence AE experience should also be assessed (Kim et al., 2002; Hwang et al., 2009; Amanzio et al., 2012). It would also be important to collect data on prior therapies that were not successful to identify those patients who have a possible history of medically unexplained complaints in the recruitment phase of RCTs that could compromise adherence to treatment. Importantly, patients who are most at risk of developing nocebo-like AEs should be identified through assessing the above-mentioned variables; for an example of a battery assessment scales refer to von Blanckenburg et al. (2013). It would be important to compare these variables in RCTs (both in the active medication group and in the placebo group) to describe potential differences in these important aspects that may be related to a possible negative treatment outcome (Amanzio et al., 2012).

In the future, it would be interesting to test in prospective RCTs the extent to which these specific variables would be related to the presence/absence of AEs with an overall approach not yet been developed, in order to take into account all these combining factors (Amanzio et al., 2012). This will help to clarify the presence of psychological distress predisposing patients to report non-specific AEs to an even greater extent (Amanzio et al., 2012). Moreover, arguing the nocebo phenomenon explicitly might help patients to become more aware of self-fulfilling prophecies induced by misattribution. In this direction, a cognitive-behavioral side effect prevention training by optimizing patients' expectations was considered a potential pathway in health care to improve a patients' quality of life during long-term medication intake (von Blanckenburg et al., 2013). This approach may be useful in patients with stabilized schizophrenia. In line with these suggestions, an assessment of the expectancies related to treatment should be better developed to give an objective measure of the individual predisposition (Younger et al., 2012). With this purpose in mind, using an additional natural history group as the trial's so-called third arm is an important factor that should be considered in RCTs (Amanzio, 2011). As to the third group, it would be possible to study the AEs because of the nocebo effects as the difference between the symptoms collected in the natural history group and the side effects presented in the placebo group (Amanzio, 2011). Indeed, natural course conditions should be incorporated more frequently in RCTs, such as in Zelen Design. This lets the disease natural history to be monitored without randomizing patients to a no treatment controls group to overcome ethical issues (Enck et al., 2013).

## Conclusions and Implications

This review of the placebo arm of RCTs of SCD patients indicates that nocebo-related AEs may be substantial. Although our research has been carefully design, some methodological shortcomings may have played an important role in the results observed. The first aspect concerns the literature search, in which potential studies have been identified. The second aspect concerns the meta-analytic experimental group heterogeneity (different for age and gender), which could be another possible confounding factor. The third aspect regards the original adopted AEs classifiers and assessment methodology. The fourth aspect—as before mentioned—concerns the meta-regression approach itself.

Because an important recognized feature of meta-analyses is that their results are critically dependent on the quality and homogeneity of the individual studies analysed (Papadopoulos and Mitsikostas, 2012), based on the general nocebo effect literature of specific neurological diseases, it is possible to suggest the inclusion of methodological assessment in clinical trial designs, which may contribute to the homogeneity of the population, and to tailor individualized treatment in patients having chronic diseases (Amanzio et al., 2016).

## Author Contributions

SP conducted studies selection, the assessment of their methodological quality and interpretation of results. She evaluated the degree of accuracy and reliability in the statistical classification. She participated in writing the article and subsequent revisions. FG conducted studies selection (*Judge 1: Cohen's kappa*), statistical analysis, and interpretation of results. He participated in writing the article and subsequent revisions. MB conducted studies selection (*Judge 2: Cohen's kappa*) and the assessment of their methodological quality. He participated in writing the article. MA conceived the study, supervise studies selection, data collection and analyses, wrote the first draft of the manuscript and subsequent revisions.

## Conflict of Interest Statement

The authors declare that the research was conducted in the absence of any commercial or financial relationships that could be construed as a potential conflict of interest.
