# Supplementary material for: Are Patients With Schizophrenia Spectrum Disorders More Prone to Manifest Nocebo-Like-Effects? A Meta-Analysis of Adverse Events in Placebo Groups of Double-Blind Antipsychotic Trials
Source: Front Pharmacol. 2019 May 17;10:502. doi: 10.3389/fphar.2019.00502 (PMC6533921; doi:10.3389/fphar.2019.00502)
Supplement: Supplementary file 1 [file Data_Sheet_1.PDF]

SUPPLEMENTARY MATERIAL

Are patients with *Schizophrenia Spectrum Disorders* more prone to manifest nocebo-like-effects? A meta-analysis of adverse events in placebo groups of double-blind antipsychotic trials

Palermo Sara [1,2#*], Giovannelli Fabio [3#], Bartoli Massimo [1], Amanzio Martina [1,2]
1 Department of Psychology, University of Turin, Turin, Italy,
2 European Innovation Partnership on Active and Healthy Ageing, Brussels, Belgium
3 Section of Psychology, Department of Neuroscience, Psychology, Drug Research, Child Health, University of Florence, Florence, Italy

# These authors share the First-Author position and equally contributed

---

* Corresponding author at:
Dr. Sara Palermo (PhD)
e-mail: sara.palermo@unito.it

Table S1: List of the studies that were excluded from the analysis (full-text selection only)

PMID	Reason for exclusion
28937706	Secondary Analysis
28750582	Not Atypical Antipsychotic
28640988	Secondary Analysis
28383362	Secondary Analysis
28320223	Other Diagnosis (no Schizophrenia Spectrum)
28277864	Secondary Analysis
28252452	Open Label
27913408	Crossover
27788310	No report of Adverse Events in the Placebo Group
27629292	Open Label
27620899	Open Label
27574838	No Report of Adverse Events
27461399	No Placebo Goup
27434314	Secondary Analysis
27379654	Crossover
27319970	Add-on (drug + placebo)
27265548	No Placebo Group
27091657	No Placebo Group
27066860	No Report of Adverse Events
27035871	No Placebo Group
26922656	Crossover
26902950	No Placebo Group
26848792	No Placebo Group
26517202	Secondary Analysis
26450657	Add-on
26431793	Open Label
26343601	No Placebo Group
26143058	Secondary Analysis
25890643	No Report of Adverse Events
25711509	Secondary Analysis
25649680	No Placebo Group
25556976	No Placebo Group
25556080	No Placebo Group
25539791	No Atypical Antipsychotic
25425069	Commentary
25281992	Secondary Analysis
25280429	Secondary Analysis
25086659	No Placebo Group
25009161	Open Label
24955752	Secondary Analysis
24925984	No Placebo Group
24870446	No Placebo Group
24846035	No Placebo Group
24794879	Secondary Analysis
24768247	Open Label
24754314	Review
24743717	No Placebo Group

24735806	No Atypical Antipsychotic
24630262	No Placebo Group
24375207	No Placebo Group
24375206	No Placebo Group
24315135	No Placebo Group
24113628	Secondary Analysis
24079855	No Placebo Group
24035633	No Report of Adverse Events
23926573	No response from the authors to the full text request
23919898	No Placebo Group
23778382	No Placebo Group
23775057	Open Label
23723707	Review
23643327	No Placebo Group
23615694	No Atypical Antipsychotic
23609405	Secondary Analysis
23583011	Secondary Analysis
23571810	No Report of Adverse Events
23559220	Open Label
23446197	Secondary Analysis
23422376	No Placebo Group
23352776	No Report of Adverse Events
23311957	No Report of Adverse Events
23218563	Secondary Analysis
23131879	No Placebo Group
24446539	No Placebo Group
23059159	No Atypical Antipsychotic
22995972	Secondary Analysis
22995933	No Placebo Group
22850268	No Placebo Group
22561475	No response from the authors to the full text request
22507686	Secondary Analysis
22489255	Other Diagnosis (no Schizophrenia Spectrum)
22475524	No Placebo Grop
22454251	No Placebo Group
22395527	No Report of Adverse Events in the Placebo Gruop
22339214	No Placebo Group
22257975	No Placebo Group
22236137	No Report of Adverse Events
22229963	Crossover
22198451	No Placebo Group
22161738	Secondary Analysis
22086749	No Placebo Group
22019076	Secondary Analysis
22000938	Secondary Analysis
21943257	Secondary Analysis
21889878	No Placebo group
21884578	No Placebo Group
21873032	Secondary Analysis
21823172	Add-on
21777507	Add-on

21775106	Secondary Analysis
21755540	No Placebo Group
21733490	Add-on
21700430	Secondary Analysis
21638329	Secondary Analysis
21568628	No Placebo Group
21549568	No Placebo Group
21508856	No Atypical Antipsychotic
21435142	No Placebo Group
21420283	No Placebo Group
21412846	No Placebo Group
21366475	No Placebo Group
21346616	Add-on
21324135	Secondary Analysis
21299844	Secondary Analysis
21294997	Others (24-hour Study)
21292928	Add-on
21277745	No Placebo Group
21240151	No response from the authors to the full text request
21209243	No Placebo Group
21200077	Others (24-hour Study)
21168920	Secondary Analysis
20961479	Secondary Analysis
20925971	Secondary Analysis
20868639	No Report of Adverse Events
20814318	No Placebo Group
20814315	No Placebo Group
20732794	No Placebo Group
20673553	No Placebo Group
20661022	Add-on
20520283	No Atypical Antipsychotic
20493663	No Placebo Group
20473064	Secondary Analysis
20457512	No Placebo group
20441726	No Placebo Group
20399985	No Placebo Group
20378185	No Placebo Group
20205074	No Report of Adverse Events in the Placebo Group
20199482	Secondary Analysis
20196185	Secondary Analysis
20155997	Commentary
20050717	No Placebo Group
20008947	No Placebo Group
19910718	Secondary Analysis
19890230	Secondary Analysis
19876039	Commentary
19843656	No Report of Adverse Events
19825908	Crossover
19822620	No Randomized Controlled Trial
19814947	Open Label
19766459	Secondary Analysis

19606529	No Placebo Group
19566763	Open Label
19560322	Secondary Analysis
19553086	No Placebo Group
19552963	Secondary Analysis
19552488	No Atypical Antipsychotic
19542525	No Placebo Group
19440083	No Placebo Group
19419595	No Placebo Group
19407274	No Randomized Controlled Trial
19323965	No Placebo Group
19269791	No Placebo Group
19269139	No Placebo Group
19230981	Secondary Analysis
19192440	No Placebo Group
19189878	Add-on
19175979	No Placebo Group
19072747	Open Label
19070995	No Placebo Group
19027269	No Placebo Group
19011427	No Placebo Group
18986646	No Placebo Group
18976782	No Placebo Group
18835660	No Placebo Group
18827289	Secondary Analysis
18801830	No Placebo Group
18794642	No Placebo Group
18790605	Secondary Analysis
18774696	No Report of Adverse Events
18700216	No Placebo Group
18626265	No Placebo Group
18575849	No Placebo Group
18568578	No Placebo Group
18545059	Secondary Analysis
18480685	Open Label
18466670	No Placebo Group
18466043	Secondary Analysis
18334911	No Report of Adverse Events
18334908	Secondary Analysis
18312044	Secondary Analysis
18303965	No Report of Adverse Events
18232726	No Placebo Group
18055944	No Placebo Group
18179662	OTHERS (cost-utility of 2nd generation antipsychotics)
18078537	No Placebo Group
18077136	No Placebo Group
18052574	No Placebo Group
18052570	No Placebo Group
18030652	No Placebo Group
18004133	No Placebo Group
18004123	No Placebo Group

17917555	No Placebo Group
17910802	Add-on
17900775	No Placebo Group
17853271	Open Label
17728106	No Placebo Group
17691076	No Placebo Group
17690599	OTHERS (small sample size)
17662577	No Placebo Group
17632222	No Placebo Group
17629731	No Placebo Group
17629725	No Placebo Group
17606658	No Placebo Group
17606657	No Placebo Group
17558641	No Placebo Group
17548746	No Placebo Group
17514185	No Placebo Group
17475386	No Placebo Group
17414740	No Placebo Group
17412473	No Placebo Group
17389899	OTHERS (genetic study)
17388705	No Placebo Group
17335319	No Placebo Group
17329466	No Placebo Group
17284138	Crossover
17284127	No Placebo Group
17224708	No Placebo Group
17151160	No Placebo Group
17126974	No Placebo Group
17107241	No Placebo Group
17090731	No Randomized Controlled Trial
17048425	No Report of Adverse Events in the Placebo Group
17029751	No Placebo Group
17015810	No Placebo Group
17010994	Secondary Analysis
16974193	Open Label
16965196	No Placebo Group
16953381	No Atypical Antipsychotic
16889453	No Placebo Group
16887334	No Placebo Group
16816798	Secondary Analysis
16783811	No Placebo Group
16754835	No Placebo Group
16648318	Other Diagnosis (no Schizophrenia Spectrum)
16601995	No Placebo Group
16585455	No Placebo Group
16540702	No Placebo Group
16426096	Secondary Analysis
16420071	Secondary Analysis
16415724	No Report of Adverse Events
16370079	No Placebo Group
16360311	No Placebo Group

16319406	No Placebo Group
16317317	No Placebo Group
16280344	Secondary Analysis
16267634	No Placebo Group
16202565	No Placebo Group
16199835	No Placebo Group
16199834	No Placebo Group
16198059	No Placebo Group
16187770	No Placebo Group
16174427	No Placebo Group
16172203	No Placebo Group
16162401	No Placebo Group
16140282	No Placebo Group
16139819	No Placebo Group
16102942	No Placebo Group
16062094	No Placebo Group
16012275	No Placebo Group
15956984	No Placebo Group
15876912	No Placebo Group
15876905	Secondary Analysis
15863811	No Placebo Group
15863797	Children
15830402	Secondary Analysis
15737251	No Placebo group
15729082	Crossover
15669892	No Placebo Group
15669890	No Report of Adverse Events
15667429	No Placebo group
15650846	Open Label
15641867	No Placebo Group
15625210	No Placebo Group
15600384	No Placebo Group
15578006	No Placebo Group
15560969	Secondary Analysis
15551194	Add-on
15551193	No Report of Adverse Events
15465981	No Placebo Group
15383183	Secondary Analysis
15380859	No Placebo Group
15328547	No Placebo Group
15298648	Add-on
15289996	No Placebo Group
15265246	Secondary Analysis
15206671	No Placebo Group
15169686	No Placebo Group
15169685	No Atypical Antipsychotics
15163258	No Placebo Group
15119920	No Placebo Group
15119916	No Report of Adverse Events
15099605	No Placebo Group
15076013	No Placebo Group

15061249	No Placebo Group
15052514	No Randomized Controlled Trial
15023570	No Atypical Antipsychotic
15003147	No Placebo Group
14754789	No Placebo Group
14751428	No Placebo Group
14751424	No Placebo Group
14744169	No Placebo Group
14733451	No Atypical Antipsychotic
14719048	Only Healthy Subjects
14687871	No Placebo Group
14658949	No report of Adverse Events
14645311	No Placebo Group
14618553	No Placebo Group
14615877	No Placebo Group
14609804	No Placebo Group
14609439	No Placebo Group
14571154	No Placebo Group
12949850	No Placebo Group
12927004	Secondary Analysis
12900300	No Placebo Group
12870569	No Placebo Group
12867218	No Placebo Group
12860365	Secondary Analysis
12827347	No Placebo Group
12823075	Other Diagnosis (no Schizophrenia Spectrum)
12811711	OTHERS (24-hour Study)
12799617	No Report of Adverse Events in the Placebo Group
12777271	No Atypical Antipsychotic
12765745	No Placebo Group
12729882	No Placebo Group
12729864	Secondary Analysis
12700715	No Placebo Group
12684609	No Placebo Group
12658913	No Randomized Controlled Trial
12606844	No Randomized Controlled Trial
12590356	No Placebo Group
12562577	No Placebo Group
12562575	No Randomized Controlled Trial
12530340	No Placebo Group
12511175	No Placebo Group
12505103	No Placebo Group
12464464	No Placebo Group
12454554	No Placebo Group
12442883	No Placebo Group
12439835	No Placebo Group
12426415	No Placebo Group
12416598	No Placebo Group
12363115	No Placebo Group
12223254	No Placebo Group
12207145	No Placebo Group

12151907	No Placebo Group
12088164	No Placebo Group
12062881	No Placebo Group
12042192	No Placebo Group
11982448	No Atypical Antipsychotic
11925292	No Placebo Group
11879174	No Randomized Controlled Trial
11874209	No Randomized Controlled Trial
11829208	No Randomized Controlled Trial
11823268	No Placebo Group
11802520	No Placebo Group
11780884	No Placebo Group
11777998	No Placebo Group
11763004	No Randomized Controlled Trial
11738537	No Placebo Group
11712620	No Placebo Group
11576036	No Placebo Group
11684748	No Placebo Group
11669086	No Placebo Group
11481167	No Placebo Group
11442893	No Placebo Group
11431240	NAEP
11329400	No Placebo Group
11291531	No Placebo Group
11247108	No Placebo Group
11232747	Secondary Analysis
11199942	Secondary Analysis
11163780	No Placebo Group
11120421	No Placebo Group
11110010	Secondary Analysis
11041534	No Placebo Group
11032464	No Placebo Group
10993126	No Placebo Group
10870870	No Placebo Group
10847315	No Placebo Group
10770466	Open Label
10722180	No Placebo Group
10721870	No Atypical Antipsychotic
10711911	No Placebo Group
10693159	No Placebo Group
10667742	No Placebo Group
10667741	No Placebo Group
10584158	Secondary Analysis
10553738	No Placebo Group
10513457	No Placebo Group
10484947	No Placebo Group
10474283	No Placebo Group
10424639	No Randomized Controlled Trial
10416728	No Placebo Group
10376128	No Placebo Group
10211147	No Placebo Group

10080557	No Placebo Group
10074877	No Placebo Group
9989566	No Placebo Group
9892302	No Placebo Group
9798076	No Placebo Group
9789907	No Randomized Controlled Trial
9777179	Crossover
9696517	No Placebo Group
9690695	No Placebo Group
9682999	No Placebo Group
9659857	No Placebo Group
9630000	No Placebo Group
9619146	No Placebo Group
9611669	No Placebo Group
9608291	No Placebo Group
9555595	No Placebo Group
9545995	No Placebo Group
9519098	No Placebo Group
9429073	No Placebo Group
9408917	No Report of Adverse Events
9406266	Secondary Analysis
9350955	No Placebo Group
9347122	No Randomized Controlled Trial
9315992	No Placebo Group
9316001	No Placebo Group
9376455	No Randomized Controlled Trial
9286184	No Placebo Group
9270900	No Atypical Antipsychotic
9298518	No Placebo Group
9376337	No Randomized Controlled Trial
9376336	No Report of Adverse Events
9262046	No Randomized Controlled Trial
9247978	No Randomized Controlled Trial
9169965	No Report of Adverse Events
9167507	Secondary Analysis
9167505	No Placebo Group
9248867	No Placebo Group
9184614	No Placebo Group
9169300	No Placebo Group
9150830	No Placebo Group
9090332	No Report of Adverse Events
9090331	No Placebo Group
9131723	No Placebo Group
9084066	No Placebo Group
9004057	No Placebo Group
9265916	No Randomized Controlled Trial
9265911	Secondary Analysis
9068769	No Report of Adverse Events
8981389	No Randomized Controlled Trial
8959478	No Randomized Controlled Trial
8942454	No Randomized Controlled Trial

8633697	No Placebo Group
8733823	No response from the authors to the full text request
8690831	OTHERS (data not available)
8834418	No Randomized Controlled Trial
8834417	No Placebo Group
8822534	No Atypical Antipsychotic
8927681	No response from the authors to the full text request
8927680	No Placebo Group
8925352	No Placebo Group
8685657	No Randomized Controlled Trial
7575103	No Placebo Group
7625474	No Placebo Group
7593706	No Report of Adverse Events
7545060	No Placebo Group
7726317	No Placebo Group
7542829	No Placebo Group
7537287	No Placebo Group
7537286	No Report of Adverse Events
7894879	No Report of Adverse Events
7802104	No response from the authors to the full text request
7884017	No Placebo Group
7858067	No Placebo Group
7872041	No Placebo Group
7806684	No Placebo Group
7986770	No Randomized Controlled Trial
7961550	No Randomized Controlled Trial
7948457	No Randomized Controlled Trial
8027415	No Placebo Group
7514366	No Atypical Antipsychotic
7520905	No Report of Adverse Events
7520904	Secondary Analysis
8267134	No Placebo Group
8267129	No Placebo Group
8115670	No Placebo Group
8048341	No Placebo Group
7509495	No Placebo Group
8260445	No Randomized Controlled Trial
7694306	No Placebo Group
8369232	Secondary Analysis
7691017	No Placebo Group
8486596	No Randomized Controlled Trial
8104468	No Randomized Controlled Trial
7683702	No Atypical Antipsychotic
8290676	No Randomized Controlled Trial
8290673	No Placebo Group
7907184	No Randomized Controlled Trial
1487623	No Placebo Group
1485530	No Placebo Group
1450186	No Placebo Group
1529745	No Report of Adverse Events
1637936	No Randomized Controlled Trial

1586270	No Placebo Group
1554036	No Placebo Group
1375801	No Placebo Group
1562863	No Placebo Group
1381102	No response from the authors to the full text request
1379883	No Placebo Group
1379882	No Placebo Group
1379877	No Placebo Group
1352521	No Placebo Group
1352050	No Report of Adeverse Events
1351405	No Placebo Group
1932400	No response from the authors to the full text request
1883257	No Placebo Group
1864575	No response from the authors to the full text request
1684310	No response from the authors to the full text request
2286708	No Placebo Group
1980827	No Placebo Group
1978858	No Randomized Controlled Trial
2378547	No Placebo Group
1973843	No Placebo Group
1978497	No Placebo Group
1978496	No Placebo Group
1978491	No Placebo Group
1978481	No Placebo Group
1978474	No Placebo Group
1978470	No Placebo Group
1978469	No Atypical Antipsychotic
1978468	No Placebo Group
1978467	No Placebo Group
1975448	No Placebo Group
1973547	No Placebo Group
2572532	No Randomized Controlled Trial
2575796	No Placebo Group
2570086	No Placebo Group
2669445	No Placebo Group
2655717	No Report of Adverse Events
2574893	No response from the authors to the full text request
2573240	No Report of Adverse Events
2570438	No Placebo Group
2480613	No Randomized Controlled Trial
3415422	No Placebo Group
3046554	No Placebo Group
3046553	No Placebo Group
2901252	No Randomized Controlled Trial
3212161	No response from the authors to the full text request
3323261	No Placebo Group
3680609	Commentary
2890671	No Placebo Group
3615572	No Placebo Group
3555385	No Placebo Group
3298328	No Placebo Group

3298419	No Placebo Group
2889495	No Placebo Group
2883680	No Placebo Group
3602321	No Placebo Group
3323023	No response from the authors to the full text request
3321153	No response from the authors to the full text request
3312398	No Placebo Group
3094627	No Report of Adverse Events
3549879	No Placebo Group
3549878	No Placebo Group
3512622	No Placebo Group
2873619	No Randomized Controlled Trial
2862139	No Placebo Group
4011672	No Placebo Group
3920705	No Placebo Group
3907278	No Placebo Group
3887439	No Placebo Group
6437365	No Randomized Controlled Trial
6736272	No Randomized Controlled Trial
6371871	No Placebo Group
6363395	No Placebo Group
6144125	No report of Adverse Events in the Placebo Group
6143579	No response from the authors to the full text request
6139394	No Placebo Group
6347119	No Randomized Controlled Trial
6356177	No Placebo Group
6349256	No Placebo Group
6135723	No Placebo Group
6419263	No Randomized Controlled Trial
6139317	No Placebo Group
6133803	No Placebo Group
6133287	No Placebo Group
6124226	No Randomized Controlled Trial
7068547	No Randomized Controlled Trial
7035441	No Placebo Group
6805002	No Placebo Group
6275811	No response from the authors to the full text request
6127747	Crossover
6126413	No Placebo Group
7030442	No Report of Adverse Events
6116246	No Placebo Group
7217025	No Placebo Group
7011249	No Placebo Group
7015789	No Placebo Group
6113618	No Placebo Group
7387340	No Placebo Group
6102458	No Randomized Controlled Trial
7352836	No Placebo Group
6768075	No Placebo Group
227340	No Placebo Group
115046	No Placebo Group

375865	No Report of Adverse Events
380268	No Placebo Group
103114	No Placebo Group
98127	No Placebo Group
209274	No Randomized Controlled Trial
354331	No Placebo Group
417377	No Randomized Controlled Trial
361345	No Placebo Group
344086	No Placebo Group
329786	No response from the authors to the full text request
326215	No response from the authors to the full text request
190970	No response from the authors to the full text request
189724	No Report of Adverse Events
11760	No Placebo Group
769722	No Placebo Group
800384	No Placebo Group
796755	No Placebo Group
1102569	No Placebo Group
1101842	No Placebo Group
241306	Crossover
4605278	No response from the authors to the full text request
17894080	No response from the authors to the full text request
17894041	No response from the authors to the full text request
4591853	No response from the authors to the full text request
4571196	No report of Adverse Events in the Placebo Group
4115602	No response from the authors to the full text request
5042825	No Placebo Group
4570652	No response from the authors to the full text request
4552130	Secondary Analysis
4939661	No Randomized Controlled Trial
4927983	No response from the authors to the full text request
4921409	No response from the authors to the full text request

Appendix 1: List of the studies that have been included in the analysis

Arato, M., O'Connor, R., Meltzer, H. Y., ZEUS Study Group. (2002). A 1-year, double-blind, placebo-controlled trial of ziprasidone 40, 80 and 160 mg/day in chronic schizophrenia: the Ziprasidone Extended Use in Schizophrenia (ZEUS) study. *Inte Clin Psychopharmacol*. 17, 207-215.

Beasley Jr, C. M., Sutton, V. K., Hamilton, S. H., Walker, D. J., Dossenbach, M., Taylor, C. C., et al. (2003). A double-blind, randomized, placebo-controlled trial of olanzapine in the prevention of psychotic relapse. *J Clin Psychopharmacol.* 23, 582-594. doi: 10.1097/01.jcp.0000095348.32154.ec

Berwaerts, J., Liu, Y., Gopal, S., Nuamah, I., Xu, H., Savitz, A., et al. (2015). Efficacy and safety of the 3-month formulation of paliperidone palmitate vs placebo for relapse prevention of schizophrenia: a randomized clinical trial. *JAMA psychiatry*. 72, 830-839. doi: 10.1001/jamapsychiatry.2015.0241

Canuso, C. M., Dirks, B., Carothers, J., Kosik-Gonzalez, C., Bossie, C. A., Zhu, Y., et al. (2009). Randomized, double-blind, placebo-controlled study of paliperidone extended-release and quetiapine in inpatients with recently exacerbated schizophrenia. *Am J Psychiatry*. 166, 691-701. doi: 10.1176/appi.ajp.2009.08040613

Casey, D. E., Sands, E. E., Heisterberg, J., & Yang, H. M. (2008). Efficacy and safety of bifeprunox in patients with an acute exacerbation of schizophrenia: results from a randomized, double-blind, placebo-controlled, multicenter, dose-finding study. *Psychopharmacology*. 200, 317-331. doi: 10.1007/s00213-008-1207-7

Cooper, S. J., Butler, A., Tweed, J., Welch, C., Raniwalla, J. (2000). Zotepine in the prevention of recurrence: a randomised, double-blind, placebo-controlled study for chronic schizophrenia. *Psychopharmacology*. *150*, 237-243.

Correll, C. U., Skuban, A., Ouyang, J., Hobart, M., Pfister, S., McQuade, R. D., et al. (2015). Efficacy and safety of brexpiprazole for the treatment of acute schizophrenia: a 6-week randomized, double-blind, placebo-controlled trial. *Am J Psychiatry*. 172, 870-880. doi: 10.1176/appi.ajp.2015.14101275

Cutler, A. J., Kalali, A. H., Weiden, P. J., Hamilton, J., & Wolfgang, C. D. (2008). Four-week, double-blind, placebo-and ziprasidone-controlled trial of iloperidone in patients with acute exacerbations of schizophrenia. *Journal of Clinical Psychopharmacology*. 28, S20-S28. doi: 10.1097/JCP.0b013e318169d4ce

Cutler, A. J., Marcus, R. N., Hardy, S. A., O'Donnell, A., Carson, W. H., & McQuade, R. D. (2006). The efficacy and safety of lower doses of aripiprazole for the treatment of patients with acute exacerbation of schizophrenia. *CNS Spectr*. 11, 691-702.

Daniel, D. G., Zimbroff, D. L., Potkin, S. G., Reeves, K. R., Harrigan, E. P., Lakshminarayanan, M., et al. (1999). Ziprasidone 80 mg/day and 160 mg/day in the acute exacerbation of schizophrenia and schizoaffective disorder: a 6-week placebo-controlled trial. *Neuropsychopharmacology*. 20, 491-505. doi: 10.1016/S0893-133X(98)00090-6

Danion, J. M., Rein, W., Fleurot, O., & Amisulpride Study Group. (1999). Improvement of schizophrenic patients with primary negative symptoms treated with amisulpride. *Am J of Psychiatry*. 156, 610-616. doi: 10.1176/ajp.156.4.610

Davidson, M., Emsley, R., Kramer, M., Ford, L., Pan, G., Lim, P., et al. (2007). Efficacy, safety and early response of paliperidone extended-release tablets (paliperidone ER): results of a 6-

week, randomized, placebo-controlled study. *Schizophr Res*. 93, 117-130. doi: 10.1016/j.schres.2007.03.003

Durgam, S., Cutler, A. J., Lu, K., Migliore, R., Ruth, A., Laszlovszky, I., et al. (2015). Cariprazine in acute exacerbation of schizophrenia: a fixed-dose, phase 3, randomized, double-blind, placebo-and active-controlled trial. *J Clin Psychiatry*. 76, e1574-82. doi: 10.4088/JCP.15m09997

Durgam, S., Earley, W., Li, R., Li, D., Lu, K., Laszlovszky, I., et al. (2016). Long-term cariprazine treatment for the prevention of relapse in patients with schizophrenia: a randomized, double-blind, placebo-controlled trial. *Schizophr Res.* 176, 264-271. doi: 10.1016/j.schres.2016.06.030

Durgam, S., Litman, R. E., Papadakis, K., Li, D., Németh, G., & Laszlovszky, I. (2016). Cariprazine in the treatment of schizophrenia: a proof-of-concept trial. *Int Clin Psychopharmacol*. 31, 61. doi: 10.1097/YIC.0000000000000110

Durgam, S., Starace, A., Li, D., Migliore, R., Ruth, A., Németh, G., et al. (2014). An evaluation of the safety and efficacy of cariprazine in patients with acute exacerbation of schizophrenia: a phase II, randomized clinical trial. *Schizophr Res.* 152, 450-457. doi: 10.1016/j.schres.2013.11.041

Egan, M. F., Zhao, X., Smith, A., Troyer, M. D., Uebele, V. N., Pidkorytov, V., et al. (2013). Randomized controlled study of the T-type calcium channel antagonist MK-8998 for the treatment of acute psychosis in patients with schizophrenia. *Hum Psychopharmacol*. 28, 124-133. doi: 10.1002/hup.2289

Fleischhacker, W. W., Hobart, M., Ouyang, J., Forbes, A., Pfister, S., McQuade, R. D., et al. (2017). Efficacy and safety of brexpiprazole (OPC-34712) as maintenance treatment in adults with schizophrenia: a randomized, double-blind, placebo-controlled study. *Int J Neuropsychopharmacol.* 20, 11-21. doi: 10.1093/ijnp/pyw076.

Gopal, S., Hough, D. W., Xu, H., Lull, J. M., Gassmann-Mayer, C., Remmerie, B. M., et al. (2010). Efficacy and safety of paliperidone palmitate in adult patients with acutely symptomatic schizophrenia: a randomized, double-blind, placebo-controlled, dose-response study. *Intl Clin Psychopharmacol*. 25, 247-256. doi: 10.1097/YIC.0b013e32833948fa

Hough, D. W., Natarajan, J., Vandebosch, A., Rossenu, S., Kramer, M., & Eerdekens, M. (2011). Evaluation of the effect of paliperidone extended release and quetiapine on corrected QT intervals: a randomized, double-blind, placebo-controlled study. *Int Clin psychopharmacol* 26, 25-34. doi: 10.1097/YIC.0b013e3283400d58

Hough, D., Gopal, S., Vijapurkar, U., Lim, P., Morozova, M., & Eerdekens, M. (2010). Paliperidone palmitate maintenance treatment in delaying the time-to-relapse in patients with schizophrenia: a randomized, double-blind, placebo-controlled study. *Schizophr Res.* 116, 107-117. doi: 10.1016/j.schres.2009.10.026

Kahn, R. S., Schulz, S. C., Palazov, V. D., Reyes, E. B., Brecher, M., Svensson, O., et al. (2007). Efficacy and tolerability of once-daily extended release quetiapine fumarate in acute schizophrenia: a randomized, double-blind, placebo-controlled study. *J Clin Psychiatry*, 68, 832-842.

Kane, J. M., Peters-Strickland, T., Baker, R. A., Hertel, P., Eramo, A., Jin, N., et al. (2014). Aripiprazole once-monthly in the acute treatment of schizophrenia: findings from a 12-week, randomized, double-blind, placebo-controlled study. *J Clin Psychiatry*. 75, 1254-1260. doi: 10.4088/JCP.14m09168

Kane, J. M., Sanchez, R., Perry, P. P., Jin, N., Johnson, B. R., Forbes, R. A., et al.(2012). Aripiprazole intramuscular depot as maintenance treatment in patients with schizophrenia: a 52-week, multicenter, randomized, double-blind, placebo-controlled study. *J Clin Psychiatry*, *73*, 617-624. doi: 10.4088/JCP.11m07530

Kane, J. M., Skuban, A., Ouyang, J., Hobart, M., Pfister, S., McQuade, R. D., et al. (2015). A multicenter, randomized, double-blind, controlled phase 3 trial of fixed-dose brexpiprazole for the treatment of adults with acute schizophrenia. *Schizophr Res.* 164, 127-135. doi: 10.1016/j.schres.2015.01.038

Kane, J. M., Zukin, S., Wang, Y., Lu, K., Ruth, A., Nagy, K., et al. (2015). Efficacy and safety of cariprazine in acute exacerbation of schizophrenia: results from an international, phase III clinical trial. *J Clin Psychopharmacol*. 35, 367-373. doi: 10.1097/JCP.0000000000000346.

Kane, J., Canas, F., Kramer, M., Ford, L., Gassmann-Mayer, C., Lim, P., et al. (2007). Treatment of schizophrenia with paliperidone extended-release tablets: a 6-week placebo-controlled trial. *Schizophr Res.*, *90*, 147-161. doi: 10.1016/j.schres.2006.09.012

Keck Jr, P., Buffenstein, A., Ferguson, J., Feighner, J., Jaffe, W., Harrigan, E. P., et al. (1998). Ziprasidone 40 and 120 mg/day in the acute exacerbation of schizophrenia and schizoaffective disorder: a 4-week placebo-controlled trial. *Psychopharmacology.* 140, 173-184.

Kinoshita, T., Bai, Y. M., Kim, J. H., Miyake, M., & Oshima, N. (2016). Efficacy and safety of asenapine in Asian patients with an acute exacerbation of schizophrenia: a multicentre, randomized, double-blind, 6-week, placebo-controlled study. *Psychopharmacol.* 233, 2663-2674. doi: 10.1007/s00213-016-4295-9

Kramer, M., Litman, R., Hough, D., Lane, R., Lim, P., Liu, Y., et al. (2010). Paliperidone palmitate, a potential long-acting treatment for patients with schizophrenia. Results of a randomized, double-blind, placebo-controlled efficacy and safety study. *Int Jof Neuropsychopharmacol.* 13, 635-647. doi: 10.1017/S1461145709990988

Kramer, M., Simpson, G., Maciulis, V., Kushner, S., Vijapurkar, U., Lim, P., et al. (2007). Paliperidone extended-release tablets for prevention of symptom recurrence in patients with schizophrenia: a randomized, double-blind, placebo-controlled study. *J Clin Psychopharmacol*. 27, 6-14. doi: 10.1097/JCP.0b013e31802dda4a

Landbloom, R., Mackle, M., Wu, X., Kelly, L., Snow-Adami, L., McIntyre, R. S., et al. (2017). Asenapine for the treatment of adults with an acute exacerbation of schizophrenia: results from a randomized, double-blind, fixed-dose, placebo-controlled trial with olanzapine as an active control. *CNS Spectr.* 22, 333-341. doi: 10.1017/S1092852916000377

Lauriello, J., Lambert, T., Andersen, S., Lin, D., Taylor, C. C., & McDonnell, D. (2008). An 8-week, double-blind, randomized, placebo-controlled study of olanzapine long-acting injection in acutely ill patients with schizophrenia. *J Clin Psychiatry*. 69, 790-799.

Lecrubier, Y., Quintin, P., Bouhassira, M., Perrin, E., & Lancrenon, S. (2006). The treatment of negative symptoms and deficit states of chronic schizophrenia: olanzapine compared to amisulpride and placebo in a 6-month double-blind controlled clinical trial. *Acta Psychiatr Scand.* 114, 319-327. https://doi.org/10.1111/j.1600-0447.2006.00887.x

Lieberman, J. A., Davis, R. E., Correll, C. U., Goff, D. C., Kane, J. M., Tamminga, C. A., et al. (2016). ITI-007 for the treatment of schizophrenia: A 4-week randomized, double-blind, controlled trial. *Biol Psychiatry*. 79, 952-961. doi: 10.1016/j.biopsych.2015.08.026

Lindenmayer, J. P., Brown, D., Liu, S., Brecher, M., Meulien, D. (2008). The efficacy and tolerability of once-daily extended release quetiapine fumarate in hospitalized patients with

acute schizophrenia: a 6-week randomized, double-blind, placebo-controlled study. *Psychopharmacol Bull*. 41, 11-35.

Loebel, A., Cucchiaro, J., Sarma, K., Xu, L., Hsu, C., Kalali, A. H., et al. (2013). Efficacy and safety of lurasidone 80 mg/day and 160 mg/day in the treatment of schizophrenia: a randomized, double-blind, placebo-and active-controlled trial. *Schizophr Res.* 145, 101-109. doi: 10.1016/j.schres.2013.01.009

Loebel, A., Silva, R., Goldman, R., Watabe, K., Cucchiaro, J., Citrome, L., et al. (2016). Lurasidone Dose Escalation in Early Nonresponding Patients With Schizophrenia: A Randomized, Placebo-Controlled Study. *J Clin Psychiatry.* 77, 1672-1680. doi: 10.4088/JCP.16m10698

Marder, S. R., Kramer, M., Ford, L., Eerdekens, E., Lim, P., Eerdekens, M., et al. (2007). Efficacy and safety of paliperidone extended-release tablets: results of a 6-week, randomized, placebo-controlled study. *Biol Psychiatry*. 62, 1363-1370. doi: 10.1016/j.biopsych.2007.01.017

McEvoy, J. P., Daniel, D. G., Carson Jr, W. H., McQuade, R. D., Marcus, R. N. (2007). A randomized, double-blind, placebo-controlled, study of the efficacy and safety of aripiprazole 10, 15 or 20 mg/day for the treatment of patients with acute exacerbations of schizophrenia. *J Psychiatric Res*. 41, 895-905. doi: 10.1016/j.jpsychires.2007.05.002

Meltzer, H. Y., Cucchiaro, J., Silva, R., Ogasa, M., Phillips, D., Xu, J., et al. (2011). Lurasidone in the treatment of schizophrenia: a randomized, double-blind, placebo-and olanzapine-controlled study. *Am J Psychiatry*. *168*, 957-967. doi: 10.1176/appi.ajp.2011.10060907

Meltzer, H. Y., Risinger, R., Nasrallah, H. A., Du, Y., Zummo, J., Corey, L., et al. (2015). A randomized, double-blind, placebo-controlled trial of aripiprazole lauroxil in acute exacerbation of schizophrenia. *J Clin Psychiatry.* 76, 1085-1090. doi: 10.4088/JCP.14m09741

Nakamura, M., Ogasa, M., Guarino, J., Phillips, D., Severs, J., Cucchiaro, J., et al. (2009). Lurasidone in the treatment of acute schizophrenia: a double-blind, placebo-controlled trial. *J of Clin Psychiatry*. 70, 829. doi: 10.4088/JCP.08m04905

Nasrallah, H. A., Gopal, S., Gassmann-Mayer, C., Quiroz, J. A., Lim, P., Eerdekens, M., et al. (2010). A controlled, evidence-based trial of paliperidone palmitate, a long-acting injectable antipsychotic, in schizophrenia. *Neuropsychopharmacology*. 35, 2072. doi: 10.1038/npp.2010.79

Nasrallah, H. A., Silva, R., Phillips, D., Cucchiaro, J., Hsu, J., Xu, J., et al. (2013). Lurasidone for the treatment of acutely psychotic patients with schizophrenia: a 6-week, randomized, placebo-controlled study. *J Psychiatr Res.* 47, 670-677. doi: 10.1016/j.jpsychires.2013.01.020

Nasser, A. F., Henderson, D. C., Fava, M., Fudala, P. J., Twumasi-Ankrah, P., Kouassi, A., et al. (2016). Efficacy, safety, and tolerability of RBP-7000 once-monthly risperidone for the treatment of acute schizophrenia: an 8-week, randomized, double-blind, placebo-controlled, multicenter phase 3 study. *J Clin Psychopharmacol.* 36, 130-140. doi: 10.1097/JCP.0000000000000479

Ogasa, M., Kimura, T., Nakamura, M., Guarino, J. (2013). Lurasidone in the treatment of schizophrenia: a 6-week, placebo-controlled study. *Psychopharmacology*. 225, 519-530. doi: 10.1007/s00213-012-2838-2

Pandina, G. J., Lindenmayer, J. P., Lull, J., Lim, P., Gopal, S., Herben, V., et al. (2010). A randomized, placebo-controlled study to assess the efficacy and safety of 3 doses of paliperidone palmitate in adults with acutely exacerbated schizophrenia. *J Clin Psychopharmacol*. 30, 235-244. doi: 10.1097/JCP.0b013e3181dd3103

Pigott, T. A., Carson, W. H., Saha, A. R., Torbeyns, A. R., Stock, E. G., & Ingenito, G. G. (2003). Aripiprazole for the prevention of relapse in stabilized patients with chronic schizophrenia: a placebo-controlled 26-week study. *J Clin Psychiatry*. 64, 1048-1056

Potkin, S. G., Cohen, M., Panagides, J. (2007). Efficacy and tolerability of asenapine in acute schizophrenia: a placebo-and risperidone-controlled trial. *J Clin Psychiatry*. 68, 1492-1500.

Potkin, S. G., Gharabawi, G. M., Greenspan, A. J., Mahmoud, R., Kosik-Gonzalez, C., Rupnow, M. F., et al. (2006). A double-blind comparison of risperidone, quetiapine and placebo in patients with schizophrenia experiencing an acute exacerbation requiring hospitalization. *Schizophr Res.* 85, 254-265. doi: 10.1016/j.schres.2006.03.027

Potkin, S. G., Saha, A. R., Kujawa, M. J., Carson, W. H., Ali, M., Stock, E., et al. (2003). Aripiprazole, an antipsychotic with a novel mechanism of action, and risperidone vs placebo in patients with schizophrenia and schizoaffective disorder. *Arch Gen Psychiatry*. 60, 681-690. doi: 10.1001/archpsyc.60.7.681

Shen, J. H., Zhao, Y., Rosenzweig-Lipson, S., Popp, D., Williams, J. B., Giller, E., et al. (2014). A 6-week randomized, double-blind, placebo-controlled, comparator referenced trial of vabicaserin in acute schizophrenia. *J Psychiatric Res*. 53, 14-22. doi: 10.1016/j.jpsychires.2014.02.012

Small, J. G., Hirsch, S. R., Arvanitis, L. A., Miller, B. G., Link, C. G. (1997). Quetiapine in patients with schizophrenia: a high-and low-dose double-blind comparison with placebo. *Arch Gen Psychiatry*. 54, 549-557.

Tandon, R., Cucchiaro, J., Phillips, D., Hernandez, D., Mao, Y., Pikalov, A., et al. (2016). A double-blind, placebo-controlled, randomized withdrawal study of lurasidone for the maintenance of efficacy in patients with schizophrenia. *J Psychopharmacol.* 30, 69-77. doi: 10.1177/0269881115620460

Truffinet, P., Tamminga, C. A., Fabre, L. F., Meltzer, H. Y., Rivière, M. E., et al. (1999). Placebo-controlled study of the D4/5-HT2A antagonist fananserin in the treatment of schizophrenia. *Am J Psychiatry*. 156, 419-425.

Tzimos, A., Samokhvalov, V., Kramer, M., Ford, L., Gassmann-Mayer, C., Lim, et al. (2008). Safety and tolerability of oral paliperidone extended-release tablets in elderly patients with schizophrenia: a double-blind, placebo-controlled study with six-month open-label extension. The *Am J of Geriatr Psychiatry*. 16, 31-43. doi: 10.1097/JGP.0b013e31815a3e7a

Van Kammen, D. P., McEvoy, J. P., Targum, S. D., Kardatzke, D., Sebree, T. B. (1996). A randomized, controlled, dose-ranging trial of sertindole in patients with schizophrenia. *Psychopharmacology*. 124, 168-175.

Table S2: Adverse events which were considered synonymous of the predefined AEs and merged with main terms

Type of predefined adverse event	Adverse events which were considered synonymous
**NERVOUS SYSTEM DISORDERS**	
Akathisia	
Ataxia	
Attention Difficulties	
Diplopia	Blurred vision
Dizziness	Lightheadedness, vertigo
Diskynetics Events	Dyskinesia
Dystonia	
Epilepsy	Convulsion
Extrapyramidal Disorders	Extrapyramidal Events
Headache	
Language difficulties	
Memory impairment	
Myalgia	
Paresthesia/Tingling	Hypoesthesia
Sedation/somnolence	Somnolence, Sedation
Tremor	
Hyperkinesia/hypertonia	hyperkinesia, hypertonia
Back pain	
Cogwheel Rigidity	
Parkinsonism	
Pain	Injection pain, pian in extremities
**PSYCHIATRIC DISORDERS**	
Abnormal thinking	aggravated schizophrenia, exacerbation of schizophrenia, paranoia, Psychiatric Disorders,
Agitation	
Aggressive reaction/behavior	Aggression, aggressiveness
Anxiety	
Apathy	
Depression	Depressive mood
Hostility	
Insomnia	Sleep disorders, sleep difficulty
Nervousness	
Psychosis	
**GASTROINTESTINAL DISORDERS**	
Abdominal Pain	Gastroenteritis
Diarrhea	Gatrointestinal upset
Dry Mouth	
Dyspepsia	
Intestine Obstruction	
Nausea	
Vomiting	
Toothache/ Tooth disorders	Toothache, tooth disorders

Note: AEs = Adverse Events
